# Pharmacological inhibition of Src kinase protects against acute kidney injury in a murine model of renal ischemia/reperfusion

**DOI:** 10.18632/oncotarget.16114

**Published:** 2017-03-10

**Authors:** Chongxiang Xiong, Xiujuan Zang, Xiaoxu Zhou, Lirong Liu, Monica V. Masucci, Jinhua Tang, Xuezhu Li, Na Liu, George Bayliss, Ting C. Zhao, Shougang Zhuang

**Affiliations:** ^1^ Department of Nephrology, Shanghai East Hospital, Tongji University School of Medicine, Shanghai 200120, China; ^2^ Department of Medicine, Rhode Island Hospital and Alpert Medical School, Brown University, Providence, RI 02903, USA; ^3^ Department of Nephrology, Shanghai Songjiang District Central Hospital, Shanghai, China; ^4^ Department of Surgery, Boston University Medical School, Roger Williams Medical Center, Boston University, Providence, RI, 02908, USA

**Keywords:** Src kinase, acute kidney injury, ischemia/reperfusion, E-cadherin, metalloproteinase

## Abstract

Activation of Src kinase has been implicated in the pathogenesis of acute brain, liver, and lung injury. However, the role of Src in acute kidney injury (AKI) remains unestablished. To address this, we evaluated the effects of Src inhibition on renal dysfunction and pathological changes in a murine model of AKI induced by ischemia/reperfusion (I/R). I/R injury to the kidney resulted in increased Src phosphorylation at tyrosine 416 (activation). Administration of PP1, a highly selective Src inhibitor, blocked Src phosphorylation, improved renal function and ameliorated renal pathological damage. PP1 treatment also suppressed renal expression of neutrophil gelatinase-associated lipocalin and reduced apoptosis in the injured kidney. Moreover, Src inhibition prevented downregulation of several adherens and tight junction proteins, including E-cadherin, ZO-1, and claudins-1/−4 in the kidney after I/R injury as well as in cultured renal proximal tubular cells following oxidative stress. Finally, PP1 inhibited I/R–induced renal expression of matrix metalloproteinase-2 and -9, phosphorylation of extracellular signal–regulated kinases1/2, signal transducer and activator of transcription-3, and nuclear factor-κB, and the infiltration of macrophages into the kidney. These data indicate that Src is a pivotal mediator of renal epithelial injury and that its inhibition may have a therapeutic potential to treat AKI.

## INTRODUCTION

Acute kidney injury (AKI) is a critical clinical syndrome caused by a variety of insults, including ischemia/reperfusion (I/R) [1, 2]. Amassing evidence confirms that AKI is associated with a high mortality rate of about 40–60% in critically ill patients [2–4]. In addition, AKI can develop into chronic kidney disease which eventually progresses into end-stage renal disease [5, 6]. The molecular mechanisms of AKI remain only partially understood, and there are no therapeutic options to alter natural history. Therefore, exploring the underlying mechanisms of AKI and seeking novel therapeutic interventions is necessary for improving its clinical outcomes.

The pathophysiology of AKI has been tightly linked to tubular epithelial cell injury and death. Although renal tubular injury and death are caused by multiple mechanisms, loss of integrity of renal parenchyma plays an essential role [7]. The integrity of intercellular junction is primarily regulated by tight junctions, which are made up of major transmembrane proteins, zonular occludins (i.e. ZO-1), claudins (i.e. claudins-1and-4) as well as adherens junctions that are composed of adherens proteins (i.e. E-cadherin). I/R injury can trigger an altered distribution and/or degradation of tight and adherens junction proteins resulting in loss of barrier function, thereby leading to back-leak and renal dysfunction [8]. As a result, disruption of tight and adherens junctions may be an early and potentially reversible therapeutic target in the treatment of AKI.

Alteration and disruption of tight and adherens junctions in ischemic AKI are regulated by several signaling pathways, including Src, which has been shown to accumulate prominently at cell-cell contact sites and focal adhesions [9–11]. Src, which is activated by phosphorylation at Tyr 416 [12], can induce tyrosine phosphorylation of some tight and adherens junction proteins such as E-cadherin and some claudins [13, 14]. Src is also shown to inhibit tight junction assembly [15, 16]. Furthermore, Src can induce the activation of other signaling pathways such as ERK1/2, STAT3 and NF-kappa B [17]. Both *in vitro* and *in vivo* studies have demonstrated that ERK1/2 activation is required for renal tubular cell apoptosis [18]. Activation of STAT3 and NF-kappa B is associated with the expression of numerous inflammatory cytokines/chemokines. Moreover, Src activity is implicated in the expression and activation of matrix metalloproteinases (MMP) 2 and 9 [19], which are upregulated in models of ischemic AKI and whose up-regulation is correlated with an increase in microvascular permeability [20, 21] and progression of AKI.

Accumulating evidence indicates that the activation of Src kinase contributes to acute injury in several organs. Paul et al first demonstrated that mice deficient in Src were resistant to ischemic injury, and administration of Src inhibitors PP1 or PP2 to wild-type animals reduced ischemic injury in the brain [22–24]; Weis et al showed that the genetic or pharmacological blockade of Src reduced edema and tissue injury following myocardial infarction [24]. Moreover, early use of a Src inhibitor reduced hepatocellular injury and enabled survival in a murine model of acute liver failure induced by azoxymethane [25]. Finally, Src tyrosine kinase inhibition prevents I/R-induced acute lung injury [26]. These data provide strong evidence that Src is implicated in the pathogenesis of acute injury to multiple organs. Pharmacological inhibition of Src has been shown to block renal epithelial cell death after cisplantin exposure through Src interaction with PKC [38, 41]. But it remains unclear whether Src is implicated in the development of AKI due to ischemia reperfusion injury.

In this study, we investigated the effect of Src inhibition on the pathogenesis of AKI in a murine model of IR-induced AKI using PP1, a selective inhibitor of Src kinase. We also examined the possible mechanisms involved in these processes.

## RESULTS

### Administration of PP1 inhibits I/R induced Src activation in the kidney

**Figure 1:**
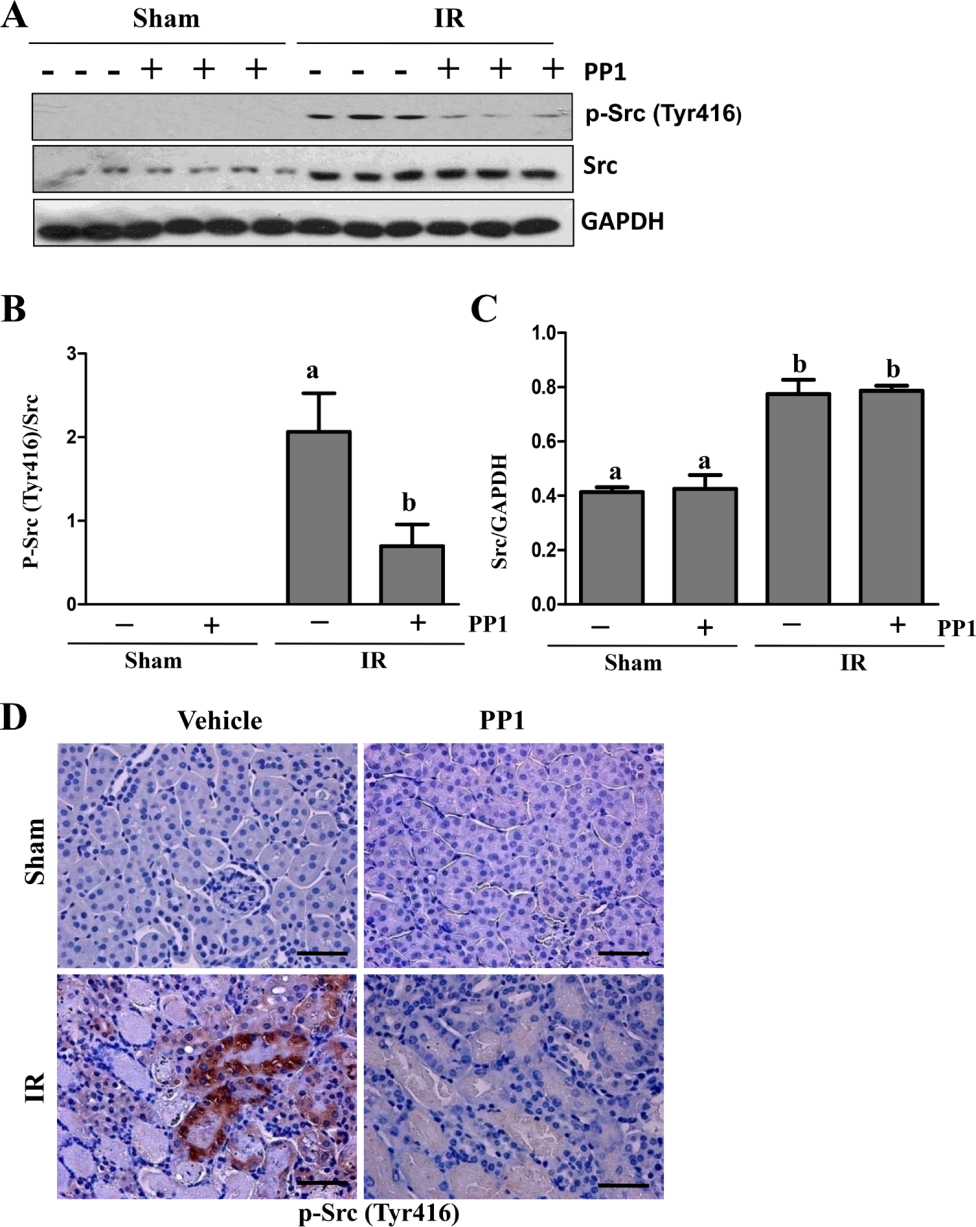
PP1 inhibits Src phosphorylation in the kidney of mice after I/R injury (**A**) Kidney lysates were subjected to Western-blot analysis with specific antibodies against P-Src (Try 416), total Src, GAPDH. (**B**) The expression level of P-Src416 was quantified by densitometry and normalized with total Src. (**C**) Total levels of Src were quantified by densitometry and normalized with GAPDH. (**D**) Representative photograph of P-Src (Tyr416) immunostaining kidney sections from sham and renal I/R injured mice. Positive staining areas are located in the renal tubules of I/R injured mice. Scale bar, 50 μm. Means with distinct letters (A, B) are significantly different from one another (*P* < 0.05).

It has been reported that Src activation occurs in the early phase of I/R-induced AKI (within 6 days) [27]. However, its role in AKI remains unclear. As a first step towards understanding the role of Src in the kidney after IR injury, we examined the effect of PP1 on the phosphorylation of Src at tyrosine 416, Immunoblot analysis revealed that phosphorylated Src at Tyr416 (p-Src) was not detectable in the sham-operated kidney, but its level was dramatically increased at 48 h after I/R injury (Figure 1A, 1B). Administration of PP1 at 2 h after the start of reperfusion significantly reduced Src phosphorylation (Figure 1A, 1B), However, PP1 treatment did not alter the expression of Src despite the increase in its basal levels after I/R injury (Figure 1C). Immunohistochemistry staining indicated that p-Src416 was primarily located in the renal proximal tubules of the injured kidney (Figure 1D), which is consistent with previous observations [27]. As the phosphorylation at tyrosine 416 positively regulates the activation of Src kinase [28], our data suggest that injury to the kidney induces Src activation in renal tubular cells and that PP1 is a potent inhibitor of Src.

### PP1 ameliorates renal dysfunction and attenuates renal damage after I/R in mice

**Figure 2:**
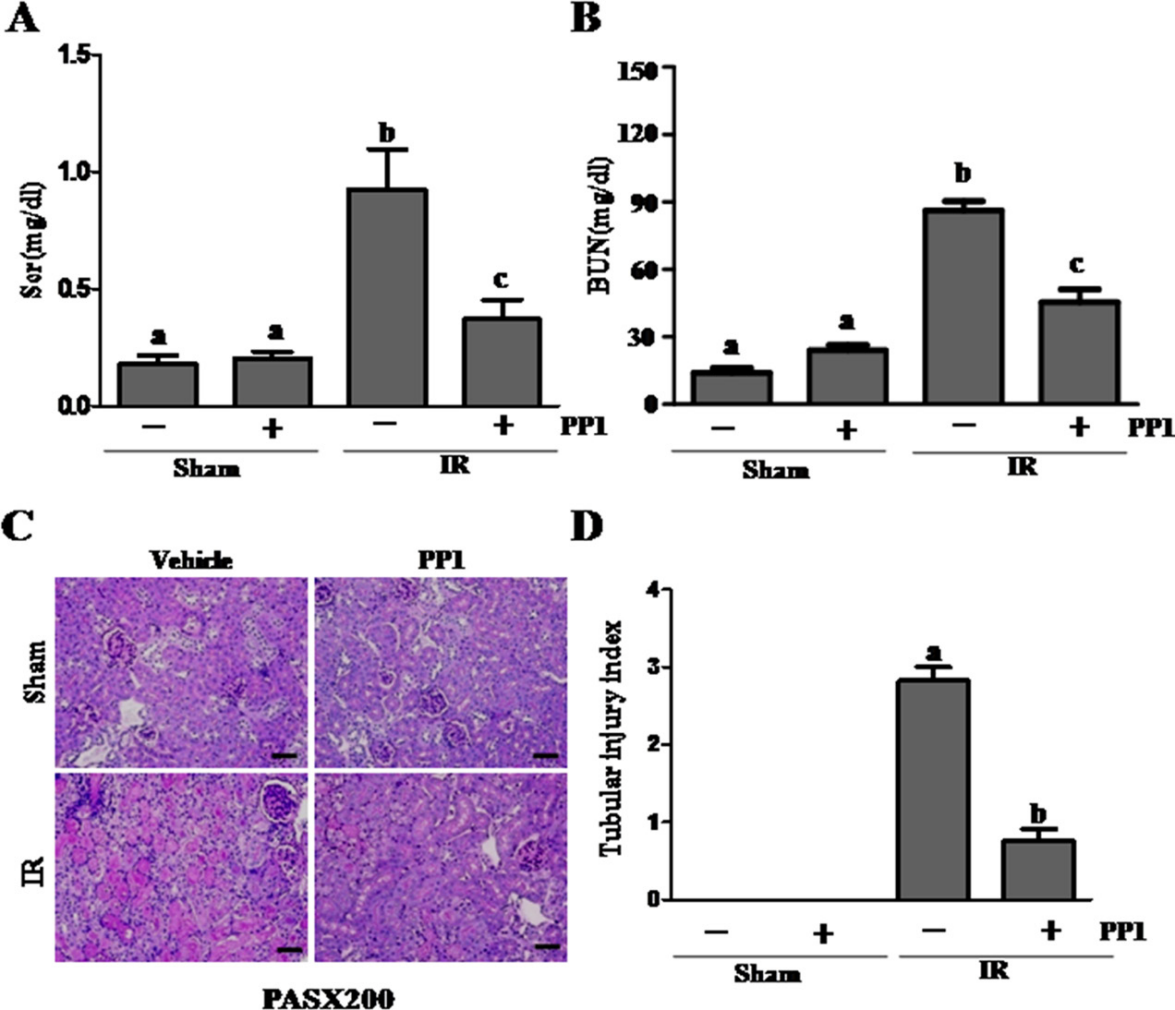
PP1 protects against renal I/R injury in mice (**A**) Plasma creatinine after renal I/R injury. (**B**) Blood urea nitrogen after renal I/R injury. (**C**) Representative image of PAS staining in kidney sections (×200). Scale bar, 50 μm. Data are represented by means ± SEM. Means with distinct letter values (A–C) are significantly different from one another (*P* < 0.05).

To examine the effect of PP1 on renal function and the pathological changes of AKI induced by I/R, we collected blood and kidney tissue samples 48 h after PP1 administration. Figure 2A and 2B show that the serum creatinine and serum blood urea nitrogen (BUN) levels significantly increased in I/R-injured mice when compared with sham-operated animals. However, administration of PP1 significantly reduced serum creatinine and BUN levels. Figure 2C and 2D show the kidney's pathological damage after I/R injury, which was characterized by tubular dilatation, swelling, necrosis, and luminal congestion. PP1treatment markedly alleviated the degree of renal tubular damage in mice subjected to I/R injury when compared with sham-operated animals. These data suggest that pharmacological inhibition of Src protects against the development of AKI following I/R injury.

### PP1inhibitsrenal tubular injury and apoptosis in the kidney

**Figure 3:**
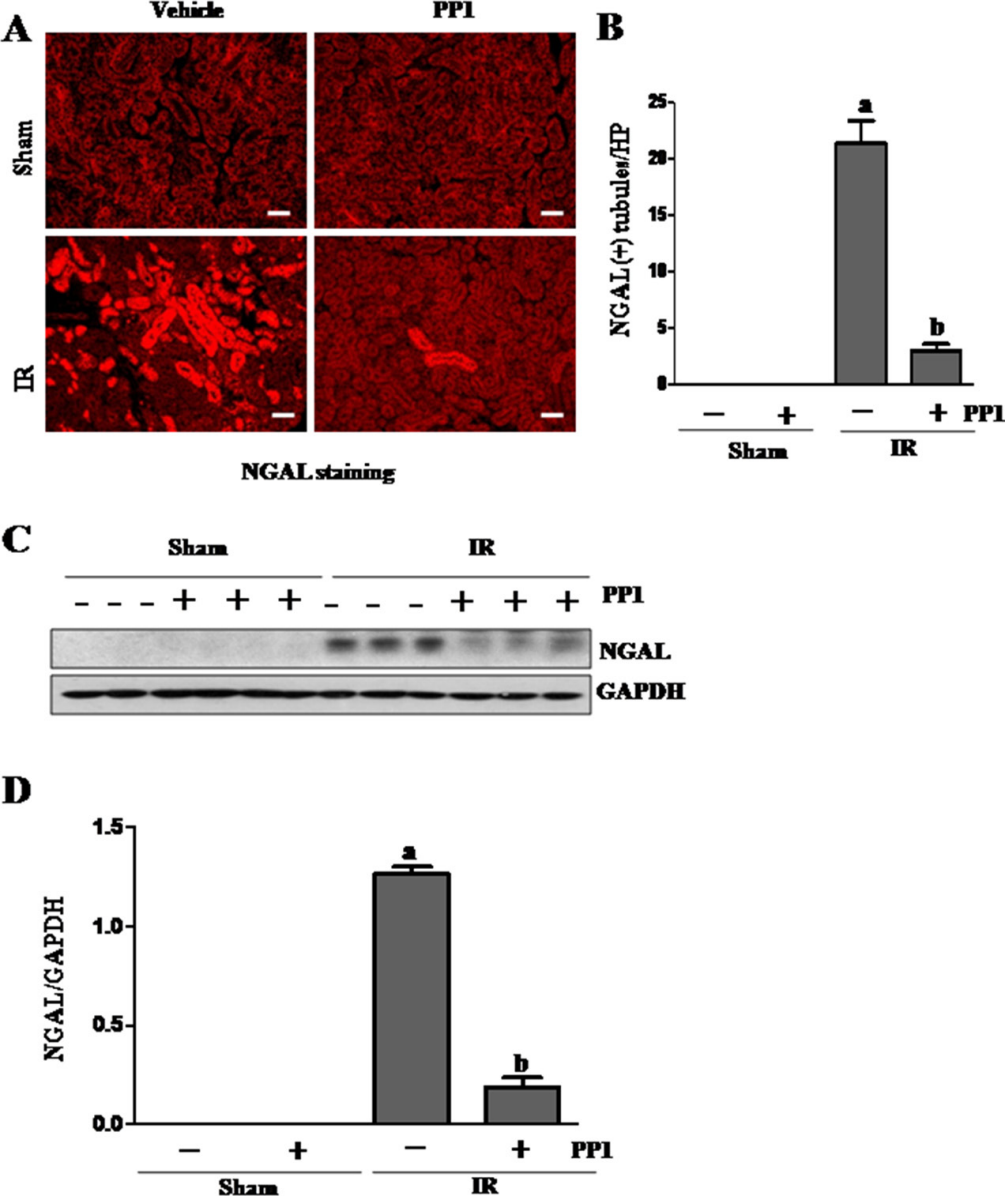
PP1 inhibits the expression of NGAL in the kidney after I/R injury (**A**) Photomicrographs (×200) display immunochemistry staining for NGAL in the kidney sections of sham and renal IRI mice. (**B**) Positive NGAL staining cells were counted in 10 high-power fields and expressed as means ± SEM. (**C**) Kidney tissue lysates were subjected to immunoblot analysis with a specific antibody against NGAL and GAPDH. (**D**) The expression level of NGAL was calculated by densitometry and normalized with GAPDH. Scale bar, 50 μm. Data are means ± SEM (*n* = 6). Means with distinct letter values (A, B) are significantly different from one another (*P* < 0.05).

**Figure 4:**
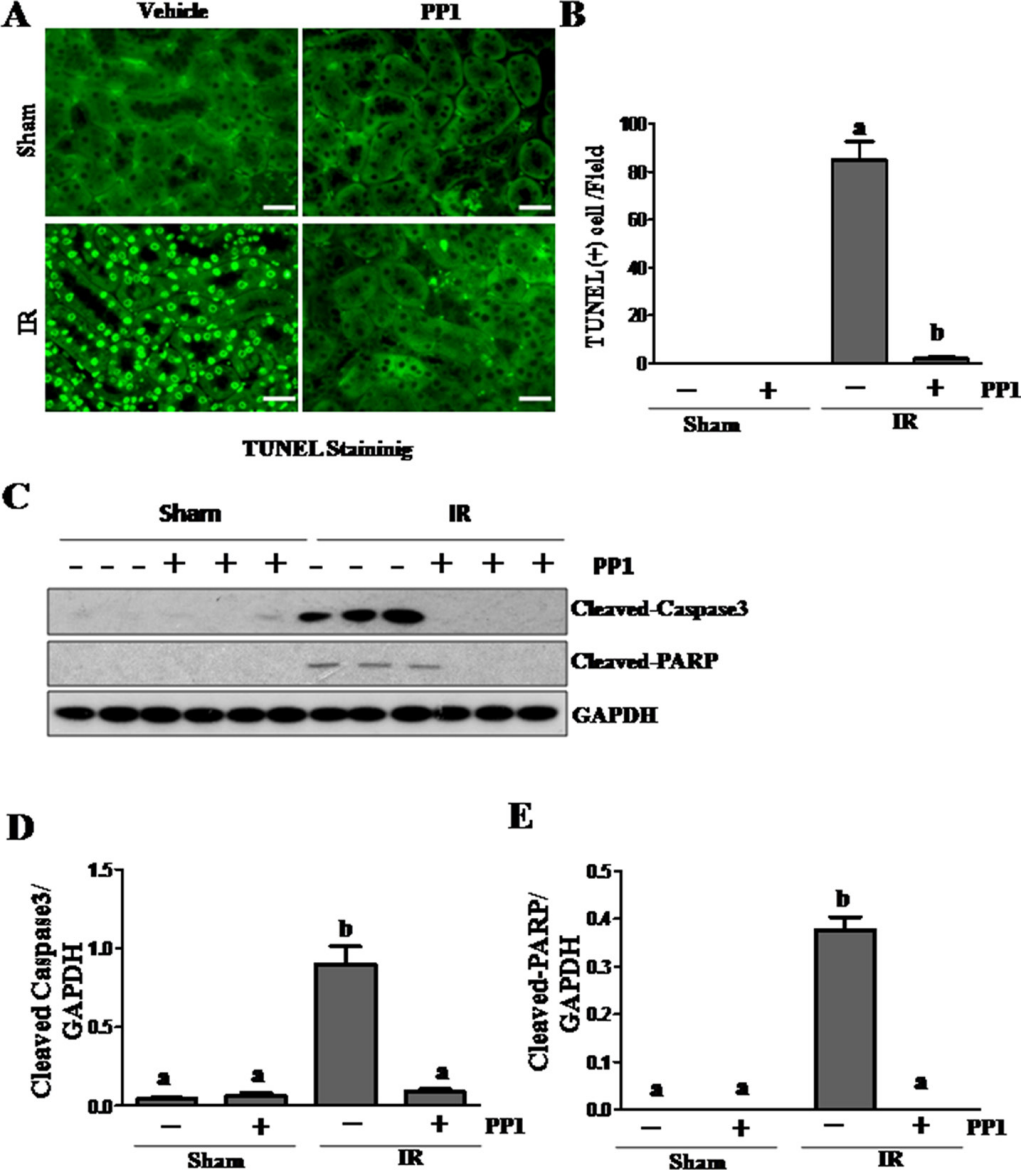
PP1 inhibits apoptosis in the kidney after I/R injury (**A**) Photomicrographs (×200) display TUNEL staining in the kidney sections of sham and renal I/R injured mice. (**B**) Positive TUNEL staining cells were counted in 10 high-power fields and expressed as means ± SEM. (**C**) Kidney tissue lysates were subjected to immunoblot analysis with specific antibodies against cleaved-caspase3, cleaved-PRAP and GAPDH. The expression level of cleaved-caspase3 (**D**) and (**E**) cleaved-PRAP were calculated by densitometry and normalized with GAPDH. Scale bar, 50 μm. Data are means ± SEM (*n* = 6). Means with distinct letter values (A, B) are significantly different from one another (*P* < 0.05).

NGAL is a well-known acute tubular damage biomarker [29, 30]. To explore the role of Src in renal tubular cell injury, we examined the effect of PP1 on NGAL expression in I/R injured kidneys via immunofluorescence staining. As shown in Figure 3, NGAL was observed in the tubules of kidney injured by I/R, but not in the sham-operated kidney (Figure 3A, 3B). PP1 treatment was shown to largely reduce the expression levels of NGAL in the injured kidney. This was confirmed by immunoblot analysis (Figure 3C, 3D). Next, we examined the effect of PP1 on renal tubular cell apoptosis using a TUNEL assay. As shown in Figure 4A, regardless of PP1 administration, TUNEL-positive cells were not detected in the sham-operated kidney; however, they were evident in the I/R injured kidney. PP1 administration significantly decreased the number of apoptotic tubular cells in the injured kidney (Figure 4A, 4B). To confirm these results, we also examined the expression of cleaved caspase-3 and cleaved PARP, two apoptosis hallmarks, via Western-blot analysis. Figure 4C–4E show that I/R injury to the kidney resulted in increased cleavage of caspase-3 and PARP, whereas PP1 treatment inhibited this response. These data further indicate that Src activation contributes to renal tubule injury and apoptosis and that PP1 treatment can ameliorate its effects.

### PP1 treatment prevents downregulation of tight and adherens junction proteins in the murine kidney after I/R injury

**Figure 5:**
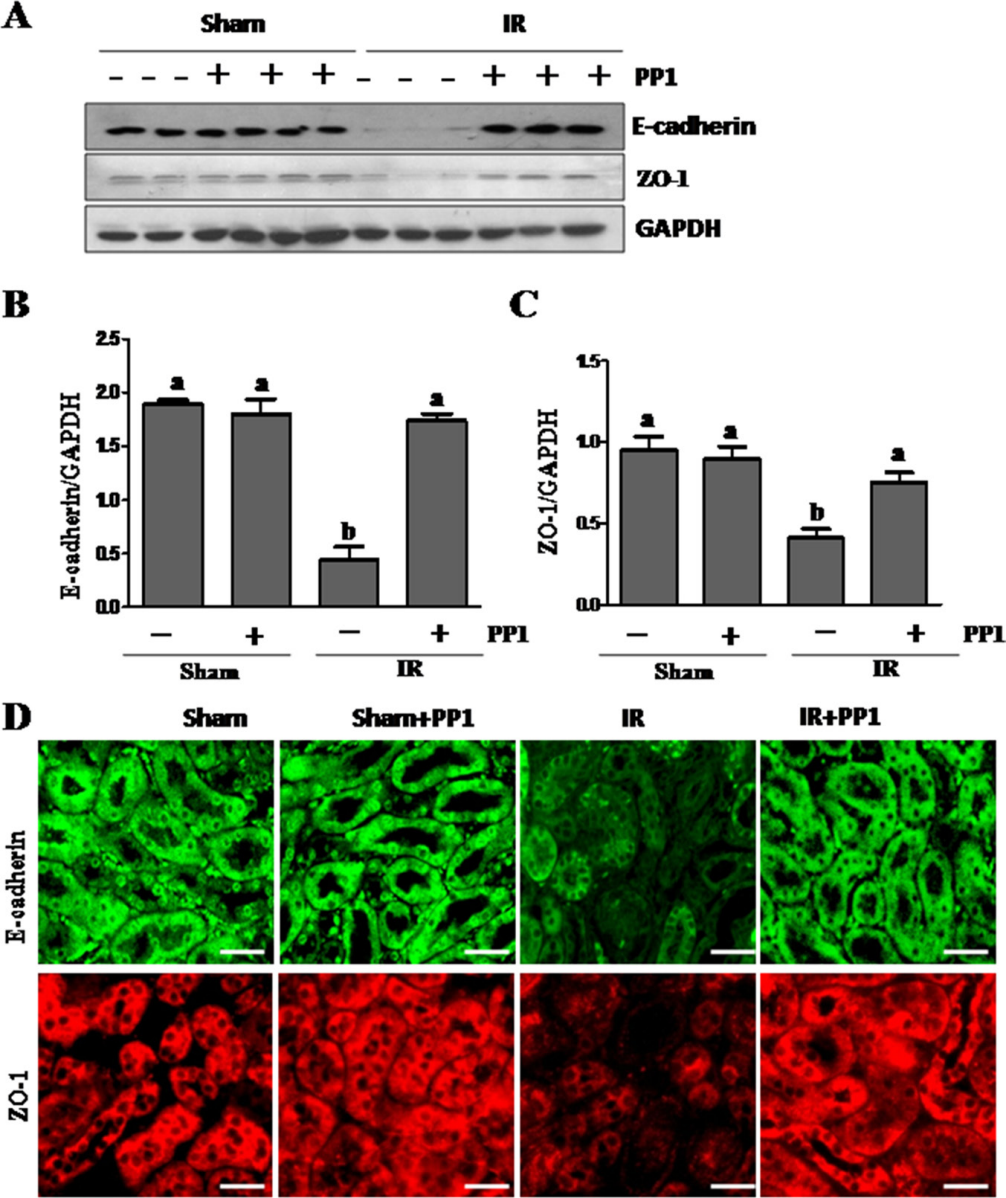
PP1 preserves the expression of E-cadherin and ZO-1 in kidney after renal IRI (**A**) Kidney lysates were subjected to western-blot analysis with specific antibodies against E-cadherin, ZO-1and GAPDH. The expression level of E-cadherin (**B**) and (**C**) ZO-1 were calculated by densitometry and normalized with GAPDH. (**D**) Representative images of immunofluorescence staining for E-cadherin and ZO-1 in kidney sections of sham and renal IRI mice. Scale bar, 50 μm. Data are means ± SEM (*n* = 6). Means with distinct letter values (A, B) are significantly different from one another (*P* < 0.05).

Recent studies have shown that cell adhesion and tight junction molecules such as the E-cadherin and ZO-1 play a crucial role in maintaining the epithelial polarity and barrier integrity necessary for the normal tubular absorption/excretion of fluid and solutes [31, 32]. Acute kidney injury results in destruction of tubular adhesion/tight junctions. To elucidate the role of Src in regulating the expression of cell adhesion and tight junction proteins, we examined the expression of E-cadherin and ZO-1 in the kidney of mice treated with and without PP1. As shown in Figure 5A–5C, I/R injured kidneys showed decreased expression levels of E-cadherin and ZO-1. These effects were inhibited by PP1 treatment. In line with these observations, immunoflunorescence staining showed that there was a significant decrease in the expression of E-cadherin and ZO-1 in I/R injured kidneys whereas PP1 treatment maintained their expression under this pathological condition (Figure 5D). Blocking Src with PP1 preserves the expression of tight junction and adhesive proteins in I/R injured kidneys.

### PP1 preserves and enhances the expression of claudin-1 and claudin-4 in the kidney after I/R injury

**Figure 6:**
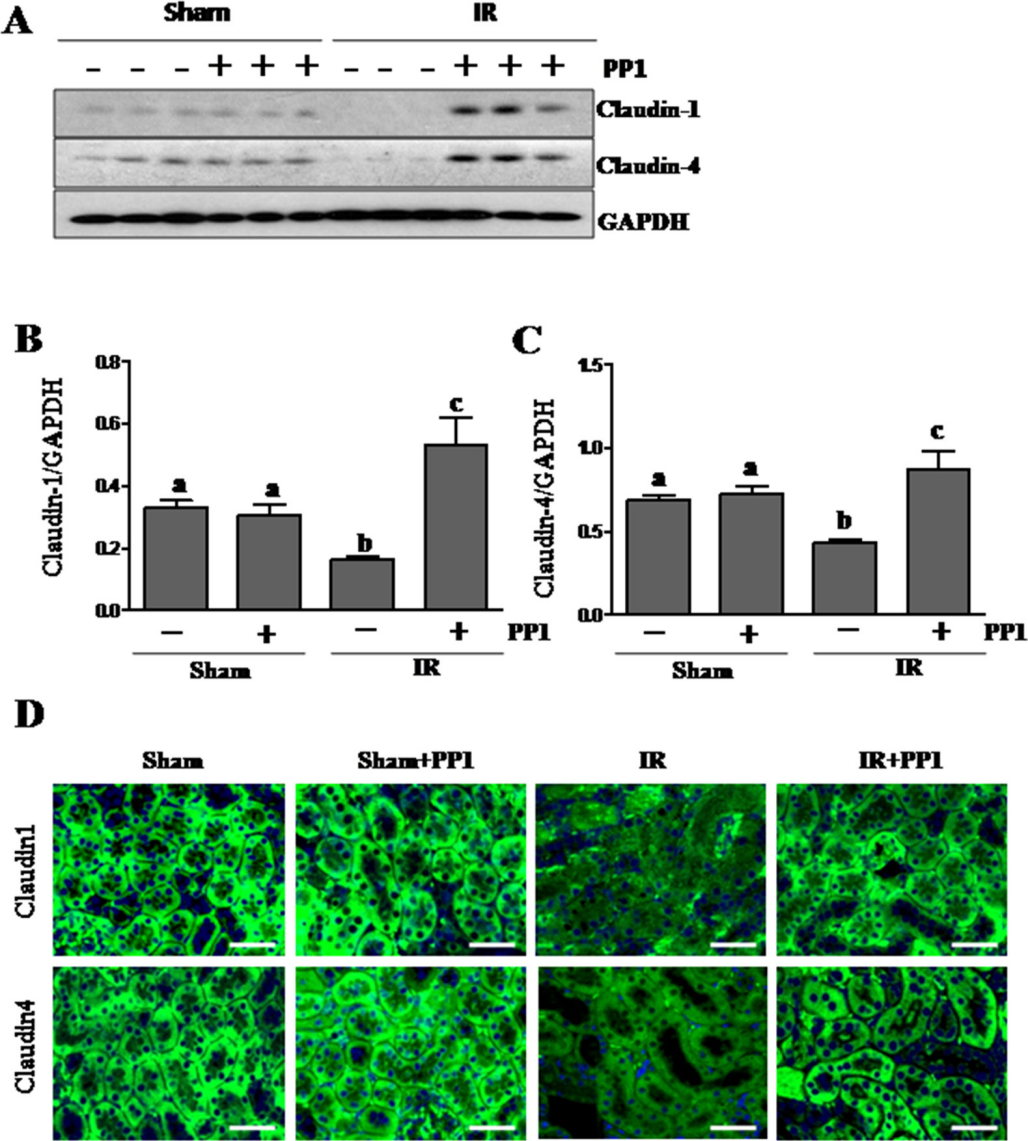
PP1 preserves the expression of claudin-1and claudin-4 in the kidney after I/R injury (**A**) Kidney lysates were subjected to Western-blot analysis with specific antibodies against claudin-1, claudin-4 and GAPDH. The expression levels of claudin-1 (**B**) and (**C**) claudin-4werecalculated by densitometry and normalized with GAPDH. (**D**) Representative images of immunofluorescence staining for claudin-1 and claudin-4 in kidney sections of sham and renal IR injured mice. Scale bar, 50 μm. Data are means ± SEM (*n* = 6). Means with distinct letter values (A, B) are significantly different from one another (*P* < 0.05).

In addition to ZO-1, other proteins including claudin-1 and claudin-4 contribute to the assembly of tight junctions [33]. As such, we examined the effect of PP1 on the expression of claudin-1and claudin-4 in the I/R injured kidney. Western-blot analysis demonstrated that I/R injury resulted in decreased expression of both claudin-1 and claudin-4 (Figure 6A–6C). Interestingly, PP1 treatment not only preserved, but also enhanced their expression. Immunofluorescence staining also revealed similar results (Figure 6D). Therefore, Src kinase also plays an important role in mediating renal expression of claudin-1 and claudin-4.

### Inhibition of Src with PP1 protects against downregulation of adhesion and tight junction proteins in cultured renal tubular epithelial cells

**Figure 7:**
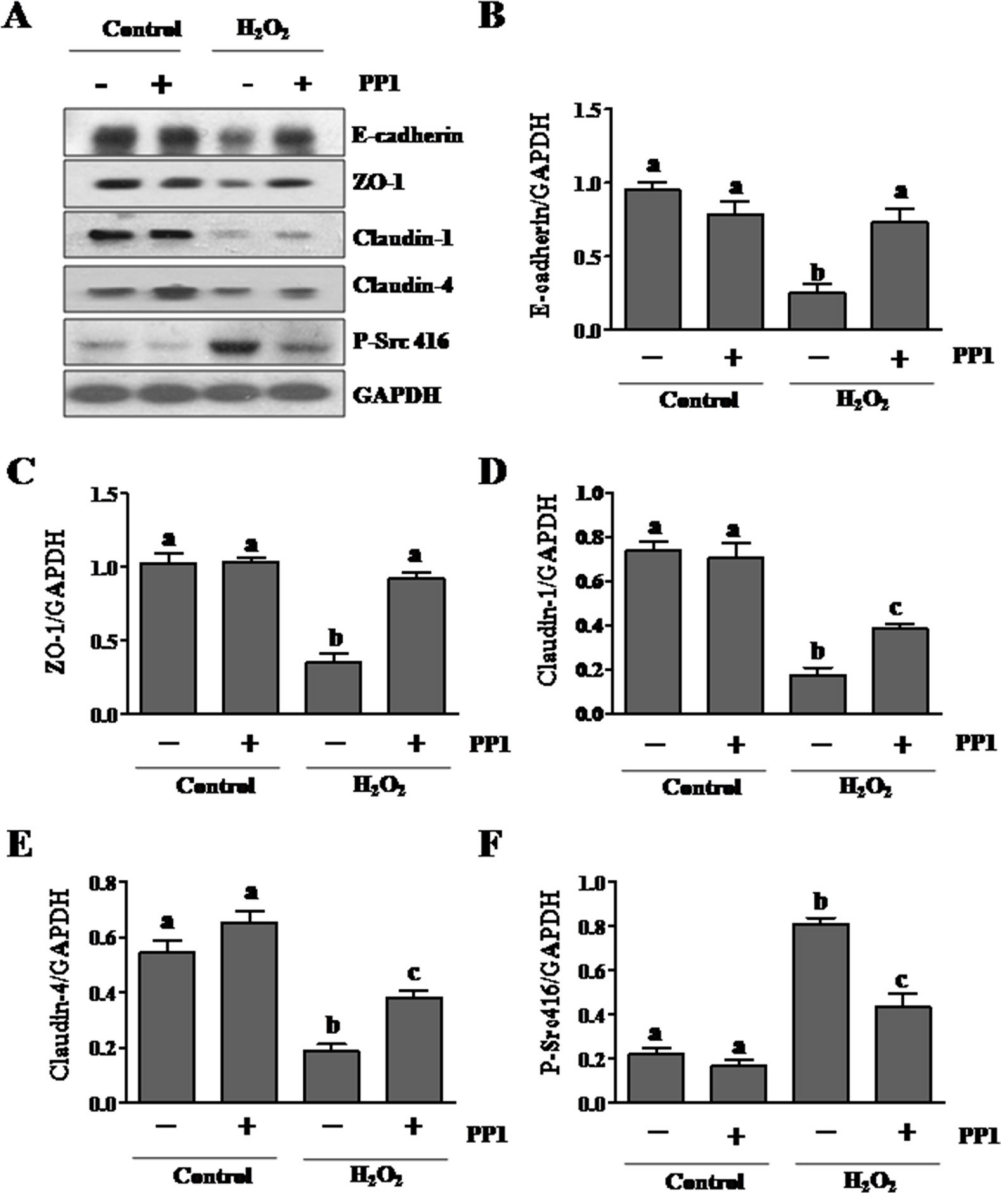
PP1 treatment preserves expression of E-cadherin, ZO-1, claudin-1 and claudin-4 in cultured renal tubule epithelial cells exposed to oxidative stress (**A**) Cells incubated in DMEM-F12 medium containing 5% FBS were exposed to PP1 (5 μM) for 24 h in the presence or absence of 1MM H_2_O_2_. Cell lysates were subject to immunoblot analysis with antibodies to E-cadherin, ZO-1, claudin, claudin4, phospho-Src (Tyr416), and GAPDH. Representative immunoblots from three or more experiments are shown. Expression levels of indicated proteins (**B**–**F**) were quantified by densitometry and normalized with GAPDH. Data are represented as the mean ± SEM. Bars with different letters (A–C) are significantly different from one another (*P* < 0.05).

To demonstrate the specific regulatory role of Src activation on adhesion and tight junction proteins in renal tubular cells, we examined the effect of PP1 on their expressions in cultured renal tubular cells exposed to oxidant injury. Exposure of renal epithelial cells to hydrogen peroxide (1 mM) resulted in decreased expression of E-cadherin (Figure 7A, 7B), ZO-1 (Figure 7A, 7C), Claudin-1 (Figure 7A, 7D), and Claudin-4 (Figure 7A, 7E), whereas PP1 treatment largely preserved expression of E-cadherin and ZO-1, and to a lesser extent, Claudin-1 and Claudin-4. PP1 pretreatment significantly inhibited the expression of P-Src (Tyr416) induced by H_2_O_2_ in this cell type (Figure 7F). Therefore, our data confirm the role of Src in mediating the downregulation of multiple adhesion and tight junction molecules in renal tubular cells.

### PP1 inhibits the expression of MMP-2 and MMP-9 in the kidney after I/R injury

**Figure 8:**
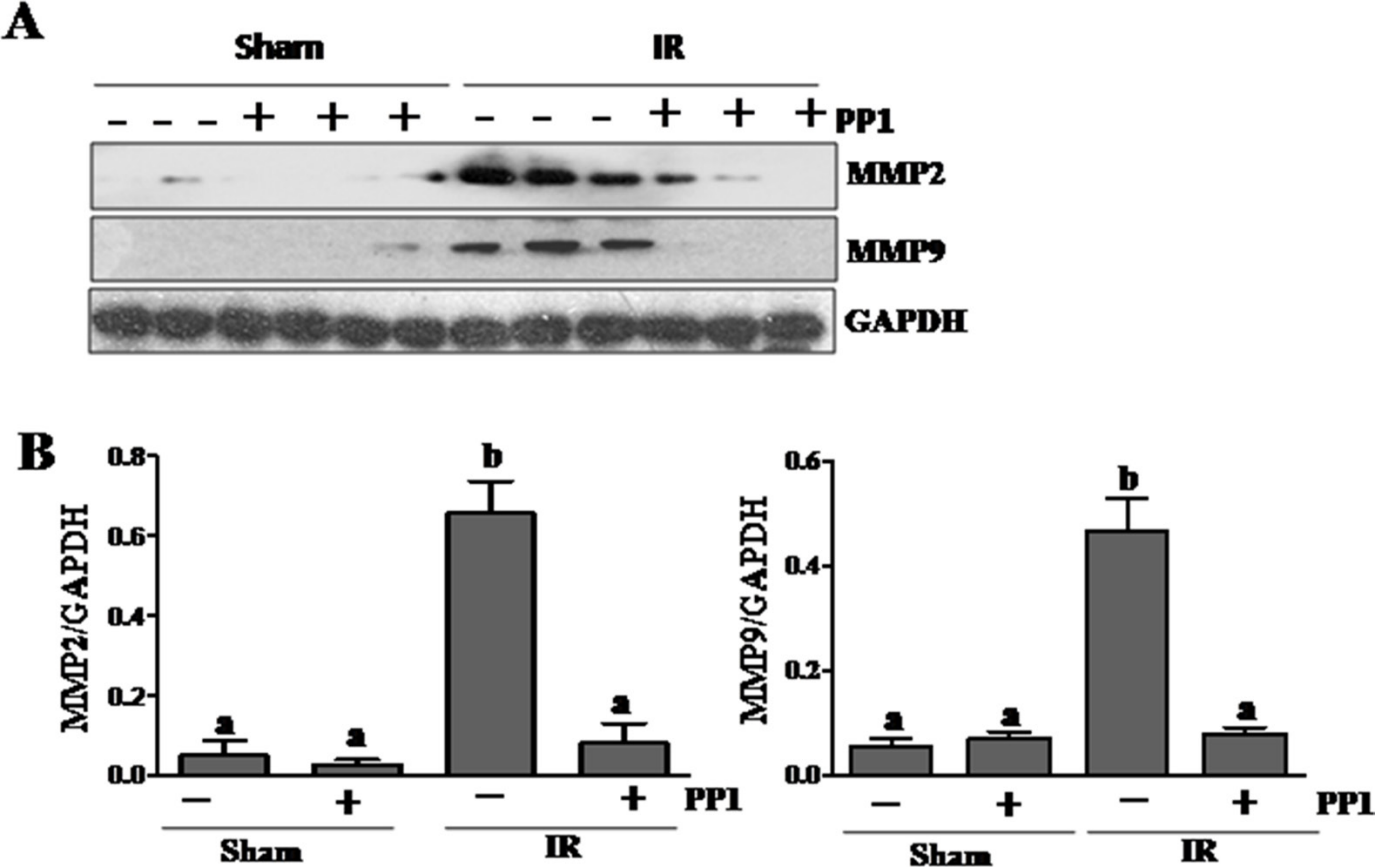
PP1 inhibits expression of MMP2 and MMP9 in the kidney after I/R injury (**A**) Kidney lysates were subjected to Western-blot analysis with specific antibodies against MMP2, MMP9 and GAPDH. The expression level of MMP2 (**B**) and MMP9 (**C**) were calculated by densitometry and normalized with GAPDH. Data are means ± SEM (*n* = 6). Means with distinct letter values (a, b) are significantly different from one another (*P* < 0.05).

Elevated MMP-2 and MMP-9 production is deleterious to epithelial barrier function [34]. To investigate the role of Src in regulating MMP-2 and MMP-9 expression in the I/R injured kidney, we examined the effect of PP1 on their expression using immunoblot analysis. Renal expression levels of MMP-2 and MMP-9 were significantly elevated following I/R injury, and PP1 treatment inhibited their expression (Figure 8A, 8B). These data suggest that Src also mediates I/R induced upregulation of MMP-2 and MMP-9 in the kidney.

### PP1 inhibits activation of ERK1/2 in the kidney after I/R injury

**Figure 9:**
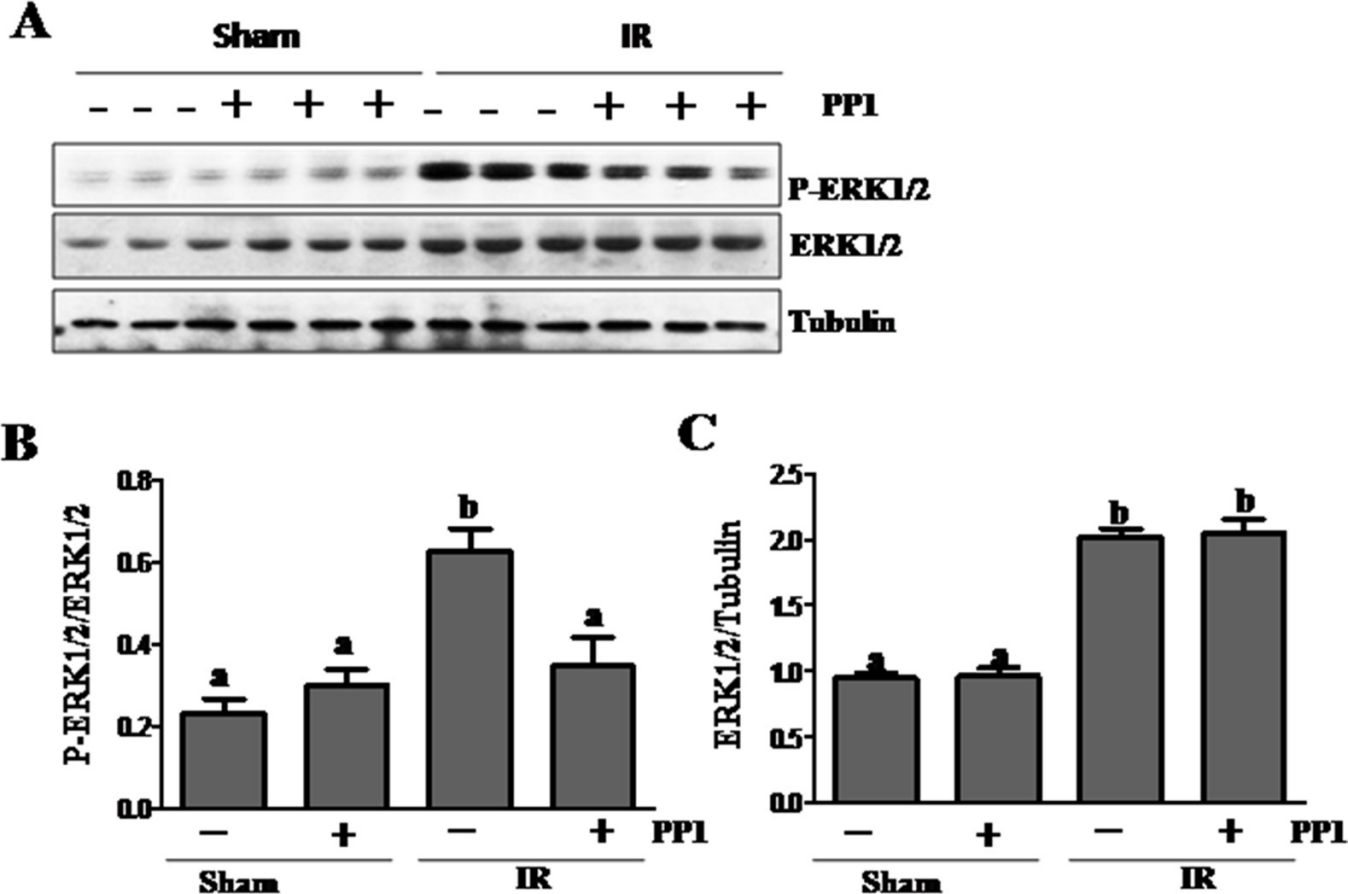
PP1 inhibitsERK1/2 phosphorylation in the kidney after I/R injury (**A**) Kidney lysates were prepared and subjected to immunoblot analysis with antibodies against phospho-ERK1/2 (pERK1/2), total ERK1/2 and tubulin. (**B**) phospho-ERK1/2 (pERK1/2) was quantified by densitometry and normalized with total ERK1/2. The total ERK1/2 (**C**) levels were quantified by densitometry and normalized with tubulin. Data are the means ± SEM. Bars with different letters (A–C) are significantly different from one another (*P* < 0.05).

Previous studies have shown that ERK1/2 mediate renal tubular cell apoptosis in animal models of cisplatin-induced AKI [18]. As a result, we examined the effect of PP1 on the activation of these kinases. As shown in Figure 9, I/R injury to the kidney resulted in increased expression of both phospho-ERK1/2 and total ERK1/2. PP1 treatment partially inhibited ERK1/2 phosphorylation without affecting their expression. Thus, Src activity is required for the activation of ERK1/2 in the I/R injured kidney.

### PP1 inhibits I/R induced phosphorylation of STAT3and NF-κB in the kidney

**Figure 10:**
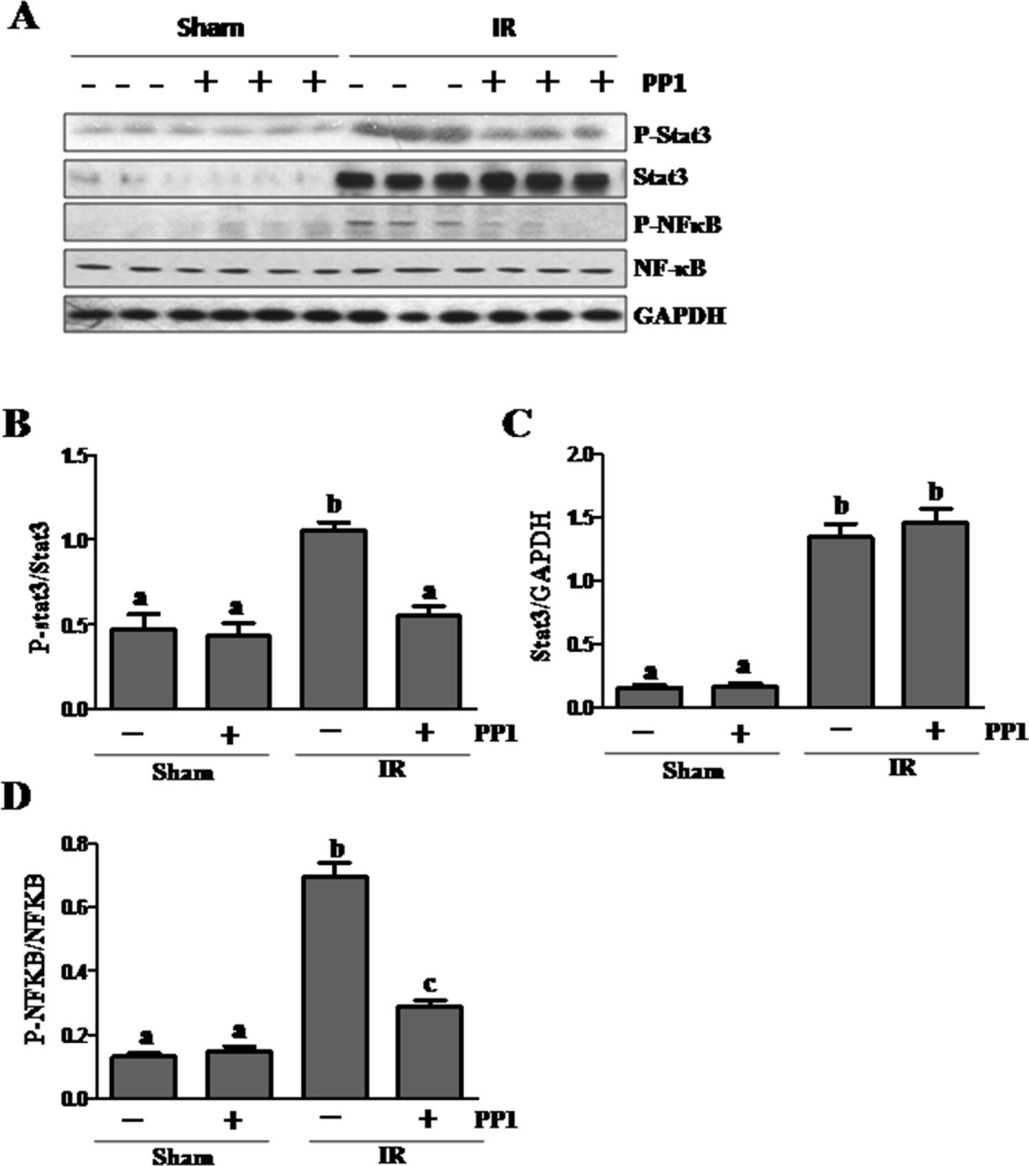
PP1 inhibits STAT3 and NF-κB phosphorylation in the kidney after I/R injury (**A**) Kidney lysates were prepared and subjected to immunoblot analysis with antibodies against phospho-STAT3 (pSTAT3), STAT3, Phospho-NF-κB, (p-NF-κB), NF-κB and GAPDH. The phospho-STAT3 (**B**) and phospho-NF-κB (**D**) were quantified by densitometry and normalized with total Stat3 or NF-κB, respectively. The total Stat3 (**C**) and NF-κB (**E**) levels were quantified by densitometry and normalized with GAPDH. Data are the means ± SEM. Bars with different letters (A–C) are significantly different from one another (*P* < 0.05).

It is well known that inflammation is critically involved in ischemic AKI [35, 36]. Since NF-κB and STAT3 are two transcription factors that mediate the expression of multiple cytokines/chemokines, we examined whether Src potentiates their activation in the kidney after I/R injury. Figure 10A–10D shows that I/R injury induced phosphorylation of STAT3 and NF-κB-p65, which was inhibited by PP1 treatment. Although the expression levels of total STAT3 and NF-κB also increased in the I/R injured kidney, PP1 treatment did not affect their expression. Therefore, Src may contribute to renal inflammation via activation of STAT3 and NF-κB signaling pathways.

### PP1 inhibits monocyte chemoattractant protein-1 expression and attenuates macrophage infiltration in I/R-induced AKI

**Figure 11:**
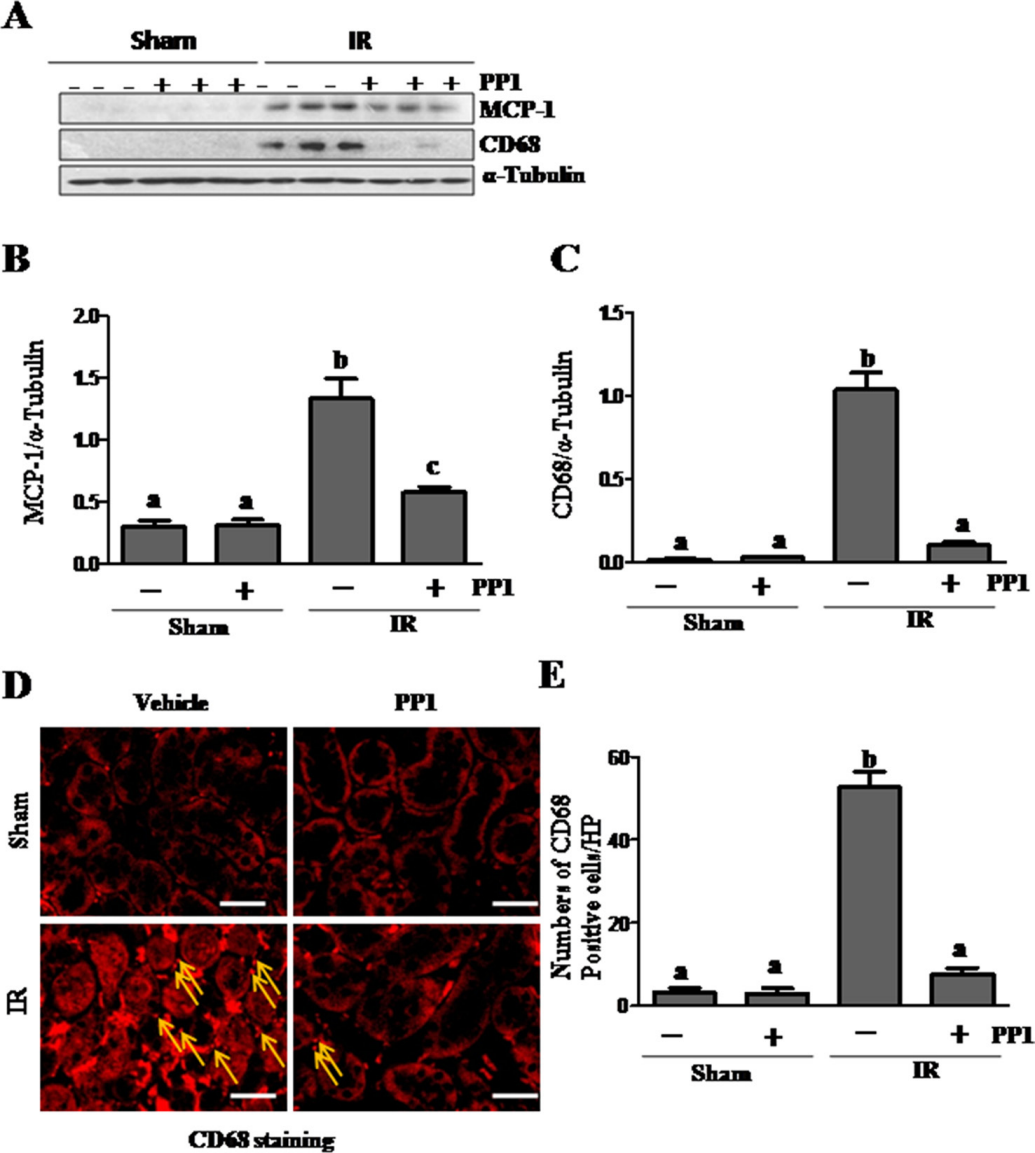
PP1 inhibits MCP-1 expression and macrophage infiltration in the kidney after I/R injury (**A**) Kidney lysates were prepared and subjected to immunoblot analysis with antibodies against MCP-1, CD68, and GAPDH. The expression levels of MCP-1 (**B**) and CD68 (**C**) were quantified by densitometry and normalized with GAPDH, respectively. (**D**) Representative images of immunofluorescence staining forCD68 in kidney sections of sham and renal I/R injured mice. Scale bar, 50 μm. Data are the means ± SEM. Bars with different letters (A, B) are significantly different from one another (*P* < 0.05).

Previous studies have shown that Src is involved in macrophage-mediated inflammatory responses [37]. To explore the role of PP1 in macrophage infiltration of the I/R injured kidney, we examined the expression of CD68 (a pan- macrophage marker) and monocyte chemoattractant protein-1(MCP-1) by Western blot analysis. As shown in Figure 11A–11C, the levels of MCP-1 and CD68 increased in the I/R kidney when compared with the sham kidney. Treatment with PP1 almost completely inhibited the expression of CD68, and to a lesser degree, MCP-1 expression. Immunofluorescence staining also showed an increase in the number of CD68-positive cells in the kidney after IR injury, while PP1 administration significantly reduced their number in the I/R injured kidney (Figure 11D, 11E). Macrophage infiltration was barely detected in the kidneys of sham-operated mice with or without PP1 treatment (D, E). These results suggest that Src plays a role in mediating expression of MCP-1 and macrophage infiltration in the I/R injured kidney.

## DISCUSSION

The inhibition of Src kinases is reported to ameliorate ischemic injury in the brain [22] and the heart [24]. In this study, we demonstrated that blocking Src kinase with PP1 also lessens the severity of renal injury, and improves renal function in a murine model of AKI induced by I/R. These protective effects of Src inhibition are associated with decreased renal tubular cell injury and apoptosis, preserved multiple adherence and tight junction protein levels, and reduced phosphorylation of transcription factors associated with the expression of inflammatory factors. On this basis, we have concluded that Src is a critical mediator of AKI and that pharmacological targeting of Src may have a therapeutic potential in the treatment of this disease.

Previous studies have shown that active Src was preferentially expressed in the S3 segment of the proximal tubule in a rat model of I/R and was detected within 6 h of reperfusion and remained elevated for at least 4 days [27]. Our study demonstrates that active Src remains elevated in the renal tubules at 48 h in a mouse model of I/R. Activation of Src in the early phase of AKI suggests that it may be involved in the development of AKI. Indeed, our data indicates that Src activation contributes to renal tubular injury and apoptosis. This was made evident by our observations that inhibition of Src with PP1 successfully inhibited expression of NGAL and reduced the number of TUNEL positive cells. PP1 treatment also blocks cleavage of caspase-3 and PARP in the injured kidney. Further, Src inhibition inhibited cleavage of caspase-3 and PARP in cultured renal tubular cells in response to oxidative stress. This is consistent with a previous observation that Src inactivation reduced renal tubular cell apoptosis in response to cisplantin treatment [38].

The mechanism by which Src inhibition protects against renal injury remains incompletely understood, but may be associated with the regulation of multiple cellular and signaling events associated with renal tubular cell death. Both our work and other studies have shown that activation of ERK1/2 contributes to apoptosis and necrosis [18, 39, 40]. In this study, we found that Src inhibition partially reduced ERK1/2 phosphorylation, which suggests a molecular basis for Src inactivation to inhibit renal tubular cell death. Nevertheless, Src may also exert its pro-apoptotic action through other mechanisms such as activation of protein kinase C delta. PKC delta has been shown to mediate apoptosis in renal tubular cells following cisplantin treatment, whereas Src is the upstream activator of PKC delta [41].

Src inhibition may also mediate renoprotection by maintaining the integrity of adherens and tight junctions in renal epithelial cells. It is well known that I/R injury can induce dissociation of cellular junctions and subsequently reduce cell-to-cell contact. This causes back leak of tubular fluid, interstitial edema and inflammation, eventually leading to cell death. Since Src has been reported to mediate oxidative-stress-induced disruption of the tight junction in Caco-2 cells [16], we examined the effect of PP1 on the expression of several proteins that are key to assembly adherens and tight junctions, including E-cadherin, ZO-1, and claudins-1/−4. Our data showed that I/R injury resulted in downregulation of all those proteins in the kidney after injury whereas PP1 treatment retained their expression both in the injured kidney and cultured renal epithelial cells. Although it currently remains unclear how Src regulates expression of each of these proteins, there are reports indicating that Src activation can induce degradation of E-cadherin and claudins [13, 14].

Nevertheless, we cannot exclude the possibility that Src kinase also causes renal injury through its effects on renal microvascular permeability. In this context, it has been demonstrated that Src mediates vascular endothelial permeability responses to TNF, reactive oxygen species, angiogenesis and vascular leakage [25–27]. Disruption of endothelial cell-cell contact [42] and alteration of cell-cell adhesion complexes [43] has also been observed in a model of ischemic AKI. On this basis, it is assumed that Src mediates both epithelial and microvascular permeability in the kidney by changing expression and/or disassembling of some proteins associated with adherens and tight junctions. Thus, it may be of interest to examine whether administration of Src inhibitors would reduce increased epithelial and microvascular permeability in a model of ischemic AKI in the future.

In addition, Src inhibition may protect against AKI through inhibition of matrix metalloproteinases (MMPs), which are zinc endopeptidases that degrade extracellular matrix and are involved in the pathogenesis of I/R [44]. It has been reported that both MMP-2 and MMP-9 are up-regulated in models of ischemic AKI and this up-regulation is correlated with an increase in microvascular permeability [20, 21]. In the current study, we found that inhibition of Src reduces expression of MMP-2 and MMP-9. Given the fact that MMP-2 and MMP-9 inhibitors significantly reduced the severity of renal tubule damage and suppressed the development of AKI after ischemia-reperfusion [44], we propose that Src may also aid in reducing epithelial and endothelial permeability and subsequent kidney damage by attenuating activity of these two proteinases. Mechanistically, Src activation has been shown to induce expression of MMP-2 and MMP-9 via AP1 and Sp1-dependent transcription [19].

Inflammation is an important component of both the initiation and extension of injury in ischemic AKI. Activation of transcription factors is required for expression of inflammatory factors. Numerous studies have demonstrated enhanced expression of NF-kappB and STAT3, two key factors in AKI [45, 46]. In this study, we demonstrated that Src inhibition suppressed phosphorylation of both factors, suggesting that Src may inhibit the production of various inflammatory factors including cytokines and chemokines. In support of this hypothesis, we demonstrated that PP1 administration inhibits expression of MCP-1, a chemokine in the injured kidney. Furthermore, blocking Src inhibited infiltration of CD68 positive macrophages to the injured area. In addition, since Src is essential for the recruitment and activation of neutrophils and other immune cells in renal ischemia reperfusion injury [47], inhibition of inflammatory responses may also be an important mechanism by which Src inhibitors protect against AKI.

Src inhibitors have shown great potential as therapeutic agents against cancer and diseases related to acute inflammatory responses. Studies from ours and other groups have demonstrated that Src mediates the pathogenesis of several chronic kidney diseases including renal fibrosis [48], diabetic nephropathy [49] and HIV-asssociated nephropathy [50]. In the past two decades, numerous Src inhibitors have been developed and some of them are under clinical trials to test their efficacy against certain cancers. Given that treatment with Src inhibitor also offers a renoprotective effect, it is speculated that Src inhibitors may be useful in the treatment of acute and chronic renal diseases. In addition, since our study showed that delayed administration of PP1 at two hours after ischemia is till effective in improving renal function and attenuating renal injury, this suggests that Src inhibitors may have a therapeutic potential for patients with AKI who are not able to come to the hospital immediately after AKI has occurred.

**Figure 12:**
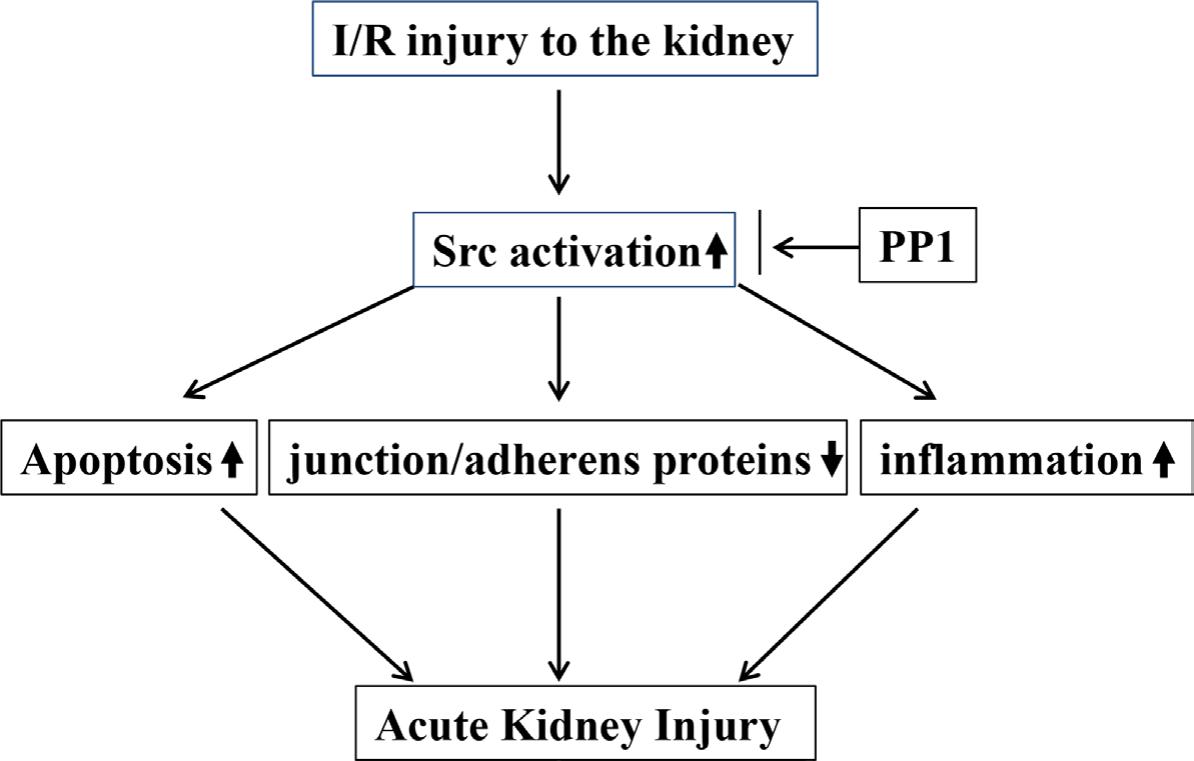
The underlying mechanism by which Src activation contributes to AKI

In summary, this study demonstrated that inhibition of Src with PP1 ameliorates pathological alterations and improves renal function following I/R injury. The mechanisms involved include suppression of renal tubular cell apoptosis, preservation of multiple junction/adheners proteins and inactivation of signaling pathways associated with renal cell death and inflammation (Figure 12). Thus, Src kinase may be a critical therapeutic target for treatment of AKI.

## MATERIALS AND METHODS

### Chemicals and antibodies

Antibodies to p-STAT3, STAT3, p-Src, Src,p-NFκBp65,NF-κBp65, cleaved-caspase-3, cleaved-PARP were purchased from Cell Signaling Technology (Danvers, MA). Antibodies to E-cadherin, CD68, MCP-1, Claudin-1, Claudin-4, MMP-2, MMP-9 and GAPDH were purchased from Santa Cruz, CA. The Tunel assay kit for apoptosis was purchased from Roche Life Science (Indianapolis, IN). Antibodies to neutrophil gelatinase-associated lipocalin (NGAL) were purchased from R&D Systems (Minneapolis, MN). Antibodies to α-tubulin, and all other chemicals were obtained from Sigma (St. Louis, MO).

### Measurement of renal function

The concentrations of the serum creatinine (Scr) and blood urea nitrogen (BUN) were determined using a creatinine assay kit (Bio Vision, Milpitas) and enzymatic assay kit (Sigma Diagnostics), respectively, according to the protocol provided by the manufacturer.

### Animal model of renal IRI and treatment protocols

The m model of I/R injury was obtained according to the procedures described in our recent studies [51]. Mice were anesthetized by ketamine (75 mg/kg, ip) and dexdomitor (50 mg/kg, im), then subjected to a flank incision on both left and right sides under sterile conditions. The bilateral renal arteries and veins were isolated from the surrounding tissue by blunt dissection and then occluded with a nontraumatic vascular clamp (85 g of pressure; RobozSurg Instruments) for 30 min at 37°C. In the sham-operated kidney, the renal pedicle was isolated but not clamped. All the mice were divided into four groups and treated with the same volume of either vehicle (DMSO) or PP1 (2 mg/kg via IP) at two hours post reperfusion. At the end of 48 h, mice were killed for collection of blood and kidneys for further analysis. All animal studies were performed according to the US Guidelines to the Care and Use of Laboratory Animals and approved by the Lifespan Animal Welfare Committee.

### TUNEL staining

The TdT-mediated dUTP nick-end labeling (TUNEL) staining for detection of apoptosis was administrated according to the protocol provided by Roches Molecular System (Branchburg, NJ). The number of TUNEL-positive nuclei per field was determined in five fields per section and five sections per kidney.

### Histology and immunofluorescent staining

The kidneys were fixed in 4% paraformaldehyde then embedded in paraffin blocks for histologic evaluation. Tissue section (3 μm in thickness) was stained with periodic acid-schiff reagent by standard protocol. Immunofluorescent staining was performed according to the procedure described in our previous studies [52]. For immunofluorescent staining, primary antibodies against NGAL (1:200), CD68 (1:150), E-cadherin (1:100), ZO-1(1:200), Claudin1 (1:200), Claudin4 (1:200)and fluorescent-conjugated secondary antibodies (1:500) were applied to the sections. The DAPI (4′,6-diamidino-2-phenylindole) staining was conducted according to the protocol provided by the manufacturer (Life Technologies, Grand Island, NY).

### Cell culture and treatments

Murine renal proximal tubular cells (TKPT) were cultured in Dulbecco's modified eagle's medium (DMEM-F12) (Sigma-Aldrich, St Louis, MO) containing 5% fetal bovine serum (FBS), 0.5% penicillin and streptomycin in an atmosphere of 5% CO2 and 95% air at 37°C. To determine the effects of PP1 on epithelial cells, PP1 was directly added to sub-confluent TKPT cells and then incubated for 24 h with or without PP1.

### Western blot analysis

Proteins of kidney lysates were analyzed by Western blot analysis as described in our previous study [52]. The densitometry analysis of our western-blot results was determined using Image J software (National Institutes of Health, Bethesda, MD).

### Statistical analysis

Data were expressed as means ± SEM for each group. Multiple-group comparison was performed using one-way analysis of variance (ANOVA). Student's *t*-test was performed to analyze the differences between two groups. *P* < 0.05 was considered statistically significant.
